# Correction: Standardized and reproducible measurement of decision-making in mice

**DOI:** 10.7554/eLife.84310

**Published:** 2022-10-27

**Authors:** International Brain Laboratory, Valeria Aguillon, Dora E Angelaki, Hannah Bayer, Niccolo Bonacchi, Matteo Carandini, Fanny Cazettes, Gaelle Chapuis, Anne K Churchland, Yang Dan, Eric Dewitt, Mayo Faulkner, Hamish Forrest, Laura Haetzel, Michael Häusser, Sonja B Hofer, Fei Hu, Anup Khanal, Christopher Krasniak, Ines Laranjeira, Zachary F Mainen, Guido Meijer, Nathaniel Miska, Thomas D Mrsic-Flogel, Masayoshi Murakami, Jean-Paul Noel, Alejandro Pan-Vazquez, Cyrille Rossant, Joshua Sanders, Karolina Socha, Rebecca Terry, Anne E Urai, Hernando Vergara, Miles Wells, Christian Wilson, Ilana B Witten, Lauren Wool, Anthony M Zador

**Keywords:** Mouse

The International Brain Laboratory, Aguillon-Rodriguez V, Angelaki D, Bayer H, Bonacchi N, Carandini M, Cazettes F, Chapuis G, Churchland AK, Dan Y, Dewitt E, Faulkner M, Forrest H, Haetzel L, Häusser M, Hofer SB, Hu F, Khanal A, Krasniak C, Laranjeira I, Mainen ZF, Meijer G, Miska NJ, Mrsic-Flogel TD, Murakami M, Noel J-P, Pan-Vazquez A, Rossant C, Sanders J, Socha K, Terry R, Urai AE, Vergara H, Wells M, Wilson CJ, Witten IB, Wool LE, Zador AM. 2021. Standardized and reproducible measurement of decision-making in mice. *eLife* **10**:e63711. doi: 10.7554/eLife.63711.

Published 20 May 2021

After publication, we realized that the text incorrectly described the quiescent period as 200-500 ms in length, whereas the mice actually experienced a quiescent period of 400-700 ms.

This error neither affects the results nor the conclusions of the article but should be corrected in order to ensure the exact replicability of the experimental design. We regret this inaccuracy and have corrected the manuscript as described below.

We have corrected the text by replacing the duration of quiescent period with the accurate duration in the two locations where it was described:

Corrected text (in Results section):

“Trials began after the mouse held the wheel still for 0.4–0.7 s”

Original text:

“Trials began after the mouse held the wheel still for 0.2–0.5 s”

Corrected text (in Materials and methods subsection, “Habituation, training and experimental protocol”):

"At the beginning of each trial, the mouse was required to not move the wheel for a quiescence period of 400–700ms (randomly drawn from an exponential distribution with a mean of 550ms).

Original text:

“At the beginning of each trial, the mouse was required to not move the wheel for a quiescence period of 200–500ms (randomly drawn from an exponential distribution with a mean of 350ms).”

The article has been corrected accordingly.

